# Functional Interaction between Adenosine A_2A_ and mGlu_5_ Receptors Mediates STEP Phosphatase Activation and Promotes STEP/mGlu_5_R Binding in Mouse Hippocampus and Neuroblastoma Cell Line

**DOI:** 10.3390/biom13091350

**Published:** 2023-09-05

**Authors:** Cinzia Mallozzi, Rita Pepponi, Lucia Gaddini, Ida Casella, Valentina Chiodi, Patrizia Popoli, Maria Rosaria Domenici

**Affiliations:** 1 Department of Neuroscience, Istituto Superiore di Sanità, 00161 Rome, Italy; 2 National Centre for Drug Research and Evaluation, Istituto Superiore di Sanità, 00161 Rome, Italy; rita.pepponi@iss.it (R.P.); lucia.gaddini@iss.it (L.G.); ida.casella@iss.it (I.C.); valentina.chiodi@iss.it (V.C.); patrizia.popoli@iss.it (P.P.)

**Keywords:** adenosine A_2A_ receptor, mGlu_5_ receptor, striatal-enriched protein tyrosine phosphatase (STEP), SH-SY5Y neuroblastoma cells, hippocampus

## Abstract

(1) Background: Recently, we found that adenosine A_2A_ receptor (A_2A_R) stimulation results in an increase in STEP phosphatase activity. In order to delve into the mechanism through which A_2A_R stimulation induced STEP activation, we investigated the involvement of mGlu_5_R since it is well documented that A_2A_R and mGlu_5_R physically and functionally interact in several brain areas. (2) Methods: In a neuroblastoma cell line (SH-SY5Y) and in mouse hippocampal slices, we evaluated the enzymatic activity of STEP by using a para-nitrophenyl phosphate colorimetric assay. A co-immunoprecipitation assay and a Western blot analysis were used to evaluate STEP/mGlu_5_R binding. (3) Results: We found that the A_2A_R-dependent activation of STEP was mediated by the mGlu_5_R. Indeed, the A_2A_R agonist CGS 21680 significantly increased STEP activity, and this effect was prevented not only by the A_2A_R antagonist ZM 241385, as expected, but also by the mGlu_5_R antagonist MPEP. In addition, we found that mGlu_5_R agonist DHPG-induced STEP activation was reversed not only by the mGlu_5_R antagonist MPEP but also by ZM 241385. Finally, via co-immunoprecipitation experiments, we found that mGlu_5_R and STEP physically interact when both receptors are activated (4) Conclusions: These results demonstrated a close functional interaction between mGlu_5_ and A_2A_ receptors in the modulation of STEP activity.

## 1. Introduction

The adenosine A_2A_ receptor (A_2A_R) is one of the four adenosine G-protein-coupled receptor subtypes (A_1_, A_2A_, A_2B_, and A_3_); it is coupled to the Gs protein (or to Golf in the striatum), and its stimulation activates adenylate cyclase, causing an increase in intracellular cAMP levels. In the central nervous system (CNS) A_2A_Rs are present at high levels in the striatum and olfactory tubercle and at lower levels in the hippocampus and cerebral cortex; they are expressed on neurons at pre- and postsynaptic levels as well as on astrocytes, microglia, and oligodendrocytes, where they modulate different physiological and pathological mechanisms, representing an interesting target for the development of new therapeutic strategies [1]. A_2A_Rs, as many other G-protein-coupled receptors, interact with other receptors, forming heteromeric complexes with unique properties and different biochemical characteristics with respect to the individual components of the heteromer. Thus, heteromeric complexes add pharmacological complexity and represent novel opportunities for drug discovery [2]. A_2A_Rs form heteroreceptor complexes with several other receptors, such as dopamine D2, cannabinoid CB1, and adenosine A_1_ receptors, as well as metabotropic glutamate 5 receptor (mGlu_5_R) [3,4,5,6].

In the hippocampus, A_2A_Rs and mGlu_5_Rs functionally interact to modulate synaptic transmission, NMDA receptor-mediated effects, and the mGlu_5_R-induced phosphorylation of the GluN2B subunit of the NMDA receptor [7,8,9]. Recently, Temido-Ferreira et al. demonstrated that the shift from LTD to LTP, considered a pathological form of synaptic plasticity that occurs in aged animals and in mouse models of Alzheimer’s disease, was corrected by an A_2A_R antagonist and a mGlu_5_R blockade [10]. Altogether, these studies suggest that the interaction between A_2A_ and mGlu_5_ receptors could be exploited as a target for therapeutic interventions for diseases in which an excessive glutamatergic tone has been demonstrated, such as Alzheimer’s disease [11].

Recently, we identified a novel role for A_2A_Rs in modulating the activation of the striatal-enriched protein tyrosine phosphatase (STEP). STEP is a brain-specific tyrosine phosphatase implicated in the pathophysiology of several neuropsychiatric diseases, and it is present in different isoforms that result from alternative splicing, with STEP46 and STEP61 representing the two isoforms that are catalytically active [12,13,14,15]. The targets of STEP include a variety of important synaptic substrates, such as kinase Fyn, AMPA, and NMDA glutamate receptors. Importantly, glutamate receptor endocytosis is regulated by STEP-mediated tyrosine dephosphorylation, thus making STEP a crucial actor in the regulation of synaptic plasticity [14]. In rodent brains and in neuronal cells, we proved that the stimulation of A_2A_Rs results in the enzymatic activation of STEP, and that the striata and hippocampi of A_2A_R-overexpressing rats show higher basal levels of STEP activation [16]. In addition, we demonstrated that this interaction between A_2A_Rs and STEP is calcium dependent and involves the calcineurin/PP1 pathway [16]. Since the activation of PKA, which results after A_2A_R stimulation, induces STEP inhibition [17], we explored in a previous study the possibility that A_2A_R could stimulate STEP activity through a direct physical interaction with STEP. We used Bioluminescence Resonance Energy Transfer (BRET) assays in SH-SY5Y cell populations, and the results suggested that STEP is probably not an A_2A_R-interacting partner [18]. Thus, the mechanism through which A_2A_R stimulation induced STEP activation seems to be indirect, possibly through the involvement of other actors. Recently, potential STEP interactors have been identified via mass spectrometry, and mGlu_5_R has been recognized as one of the 315 candidate proteins that could interact with STEP [19]. Given the well-known interaction between A_2A_ and mGlu_5_ receptors [7,20,21,22], this finding suggests mGlu_5_R as a possible mediator of A_2A_R effects on STEP activity. Indeed, the stimulation of mGlu_5_R increased STEP translation at dendritic levels and AMPA receptor endocytosis, a mechanism that could be involved in DHPG-induced LTD [12,23,24,25]. Noteworthy, mGlu_5_R is coupled with Gq proteins and its stimulation induced PLC activation and intracellular calcium increase [26], thus representing a good candidate for mediating the calcium-dependent effects of A_2A_R on the activity of STEP. Therefore, the objective of this study was to verify the involvement of mGlu_5_Rs in A_2A_R-induced STEP activation. To this aim, we first evaluated whether A_2A_ and mGlu_5_ receptors interact to modulate STEP activity in the SH-SY5Y neuroblastoma cell line and in mouse hippocampal slices and then verified the direct binding of endogenous STEP to endogenous mGlu_5_R via co-immunoprecipitation experiments (Co-IP).

## 2. Materials and Methods

### 2.1. Drugs

We obtained 2-p-(2-Carboxyethyl)phenethylamino-5′-N-ethylcarboxamidoadenosine hydrochloride hydrate (CGS 21680) from Sigma-Aldrich (Merk Life Science, Milan, Italy); (S)-3,5 Dihydroxyphenylglycine (DHPG), 4-(2-[7-Amino-2-(2-furyl)[1,2,4]triazolo [2,3-a][1,3,5]triazin-5-ylamino]ethyl)phenol (ZM 241385), and 2-Methyl-6-(phenylethynyl) pyridine hydrochloride (MPEP) were obtained from Tocris (Bio-Techne, Milan, Italy).

DHPG was dissolved in H_2_O. All the other drugs were dissolved in DMSO, and the maximum final percentage in all the treatments did not exceed 0.01%. The antibodies monoclonal anti-STEP (23E5), polyclonal anti-STEP and monoclonal anti-mGluR_5_ (D6E7B) were purchased from Cell Signaling Technology (Danvers, MA, USA); polyclonal anti-mGlu_5_R was obtained from Millipore (Temecula, CA, USA). Protein A/G PLUS agarose was obtained from Santa Cruz Biotechnology (Dallas, TX, USA); para-nitrophenyl phosphate (p-NPP) was obtained from Sigma-Aldrich (St. Louis, MO, USA).

### 2.2. Animals

C57Bl/6 mice were used. The animals were kept under standardized temperature (22 °C), humidity (55%), and lighting conditions (on a 12 h light/dark cycle), with water and food ad libitum, in standard cages (56 × 38 × 20 cm, two animals per cage). All animal procedures were carried out according to the principles and procedures outlined in the European Community Guidelines for Animal Care, DL 26/2014, via the application of the European Communities Council Directive, 2010/63/EU, and the FELASA and ARRIVE guidelines. All animal procedures were approved by the Italian Ministry of Health (code n. 1191/2020–PR) and by the local Institutional Animal Care and Use Committee (IACUC) at Istituto Superiore di Sanità (Rome, Italy). We used a total of 20 mice, both males and females, between 2 and 3 months of age.

### 2.3. Preparation of Mouse Hippocampal Slices and Treatment

To obtain hippocampal slices, the mice were decapitated between 9:00 a.m. and 12:00 a.m. under isofluorane anesthesia; the brains were removed from the skulls, and the hippocampi were isolated. With the use of a tissue chopper (McIlwain), both hippocampi were cut to obtain transverse slices (300 µm). The slices were then incubated for at least 1 h in artificial cerebrospinal fluid (ACSF) containing (in mM): 126 NaCl, 3.5 KCl, 1.2 NaH_2_PO_4_, 1.2 MgCl_2_, 2 CaCl_2_, 25 NaHCO_3_, and 11 glucose (pH 7.3) saturated with 95% O_2_ and 5% CO_2_. The slices were treated for 20 min with CGS 21680, 300 nM or for 10 min with DHPG 100 µM. ZM 241385 (500 nM) and MPEP (10 µM) were added 15 min before and then kept throughout the application of CGS 21680 or DHPG. The concentrations of A_2A_R ligands were chosen on the basis of a previous paper in which these concentrations proved to be effective in modulating STEP activity [16]. As for the mGlu_5_R ligands, we obtained the concentrations from the literature [24,27].

### 2.4. SH-SY5Y Cell Culture and Treatment

SH-SY5Y human neuroblastoma cells (Sigma-Aldrich, St. Louis, MO, USA, from The European Collection of Authenticated Cell Cultures, ECACC, Public Health England) were used. This cell line is not listed as a commonly misidentified cell line by the International Cell Line Authentication Committee (ICLAC; http://iclac.org/databases/cross-contaminations/, on 16 January 2023) and was not further authenticated during the last five years. The SH-SY5Y cells were grown in Dulbecco’s Modified Eagle Medium plus F12 in a 1:1 ratio, containing 10% bovine serum, 1% L-glutamine, and 1% penicillin–streptomycin (all from Euroclone, Italy); they were maintained at 37 °C in a humidified 5% CO_2_ atmosphere and used within passage 30. For the Western blot (WB) and Co-IP experiments, the cells were seeded in six-well plates at a density of 500,000/4 mL/well. Under these conditions, the cells are not differentiated into neurons. Twenty-four hours after the onset of the culture, the cells were treated for 5 min with CGS 21680 (300 nM) or DHPG (10 µM). ZM 241385 (500 nM) and MPEP (10 µM) were applied 20 min before and then along with CGS 21680 or DHPG. The SH-SY5Y cells were maintained at 37 °C under 5% CO_2_ for the duration of the experiments.

### 2.5. cAMP Measurements in SH-SY5Y Cell Culture

A cAMP assay was performed as described in Vezzi et al. [28]. Briefly, SH-SY5Y cells previously engineered for the stable expression of the cAMP GloSensor-22F probe (purchased from Promega), which had been seeded a day before into 96-well white plates (Packard), were washed once with PBS and incubated for 60 min in 50 μL of PBS containing 25 mM of glucose and 2 mM of luciferin (Oz Bioscience). Next, 50 μL of PBS containing 100 μM of rolipram with increasing concentrations of CGS 21680, alone or in combination with ZM 241385 at 1 nM, 10 nM, or 100 nM, was added to the wells. The plates were immediately transferred into a luminometer (Victor Light, PerkinElmer, Waltham, MA, USA). The total luminescence in each well (counts per second) was recorded at 30 s intervals for 60 min with an integration time of 0.5 s. All data were fitted using a general logistic function, as described in [28].

### 2.6. STEP Activity Assay on Immunoprecipitates

The SH-SY5Y neuroblastoma cells and mouse hippocampal slices were solubilized in RIPA buffer (in mM: 100 Tris-HCl, pH 7.5, 600 NaCl, 4% (*w*/*v*) Triton X-100, 4% (*v*/*v*) sodium deoxycholate, 0.4% sodium dodecyl sulfate (SDS) (*v*/*v*), 0.4 PMSF), and protease inhibitors (Complete, Roche Diagnostics (Basel, Switzerland), syringed, and kept on ice for 1 h. After centrifugation at 10,000× *g* for 10 min at 4 °C, the supernatant was incubated with 50% (*w*/*v*) protein A/G PLUS agarose beads for 2 h at 4 °C and clarified via centrifugation. The samples (1 mg of protein/mL) in a volume of 1 mL were incubated overnight at 4 °C in a rotating wheel with a monoclonal anti-STEP antibody (2 μg/sample). The immunocomplex was precipitated via the addition of 50% (*w*/*v*) Protein A/G PLUS agarose beads. To measure the activity of STEP, the immunoprecipitates were extensively washed and suspended in 200 μL of assay buffer (in mM: 25 Tris-HCl, pH 7.4, 20 MgCl_2_, and 0.1 PMSF) containing 15 mM of p-NPP and incubated 60 min at 37 °C under gentle stirring. The phosphatase activity of STEP was measured in the clarified supernatants via the colorimetric quantitation of the formation of p-nitrophenol at 410 nm, using a spectrophotometer.

### 2.7. Co-Immunoprecipitation (Co-IP) Assay and Western Blot (WB) Analysis

The Co-IP protocol requires gentle assay conditions during immunoprecipitation to maintain the protein–protein interaction. The SH-SY5Y cells or hippocampal slices were solubilized in a Co-IP-modified RIPA buffer (25 mM of Tris-HCl at a pH of 7.4, 150 mM of NaCl, 1% TritonX-100, 0.5% Na-deoxycholate, 1 mM of EDTA, 1 mM of MgCl_2_, and 1 mM of PMSF) with the addition of a complete cocktail inhibitor (Roche). The lysates were syringed and kept on ice for 1 h. After centrifugation at 10,000× *g* for 10 min at 4 °C, the supernatants were incubated with 50% (*w*/*v*) protein A/G PLUS agarose beads for 2 h at 4 °C and clarified via centrifugation. The samples (1 mg of protein/mL) in a volume of 1 mL were incubated overnight at 4 °C in a rotating wheel with a monoclonal anti-STEP (2 µg/sample) or polyclonal anti-mGlu_5_R antibodies (1 μg/sample). The immunocomplex was precipitated via the addition of 50% (*w*/*v*) Protein A/G PLUS agarose beads, and the presence of bound protein in the immunocomplexes was revealed via WB. For the WB analysis, the protein samples were separated using gradient (4–12%) pre-casted gels (Thermo Scientific, Waltham, MA, USA), and the proteins were transferred to nitrocellulose using the Trans Blot Turbo system (Bio-Rad, Hercules, CA, USA). The bots were washed with Tris-buffered saline (TBS) 0.05% and Tween 20 (TTBS) and blocked with 5% BSA in TTBS for 2 h. The washed nitrocellulose filters were incubated overnight at 4 °C with the appropriate antibody. After extensive washes in TTBS, the immunoreactive bands were detected via chemiluminescence coupled to peroxidase activity (ECL kit; Euroclone, Milano, Italy) and quantified using a Bio-Rad ChemiDoc XRS system.

### 2.8. Statistical Analysis

Statistical analyses were performed with GraphPad Prism software version 6.07 (San Diego, CA, USA). The results are expressed as mean values ± standard errors of the mean (SEM) and were analyzed with a one-way ANOVA test followed by Tukey’s multiple comparisons test. Significance was accepted at *p* ≤ 0.05.

## 3. Results

### 3.1. Metabotropic mGlu_5_ and Adenosine A_2A_ Receptors in the SH-SY5Y Neuroblastoma Cell Line

Before performing experiments in the SH-SY5Y cells, we checked for the presence of mGlu_5_ and A_2A_ receptors in this cell line. Since the levels of mGlu_5_R in the SH-SY5Ycell line has never been described, we performed a Western blot analysis and demonstrated that mGlu_5_R is clearly expressed by the cells (Figure S1). As the expression of A_2A_R in SH-SY5Y cells is well documented [29], we verified the integrity of A_2A_R signaling by using an SH-SY5Y cell line stably espressing a cAMP luminescent probe. We measured cAMP levels in response to increasing concentrations of the A_2A_R agonist CGS 21680. As expected, we found that CGS 21680 dose dependently increased cAMP levels, and that the presence of increasing concentrations of the A_2A_R antagonist ZM 241385 produced a rightward shift of the agonist curve, thus confirming that the SH-SY5Y cells endogenously expressed functional A_2A_R (Figure S2).

### 3.2. Adenosine A_2A_Rs Modulate STEP Activity through the Involvement of mGlu_5_Rs

**Figure 1:**
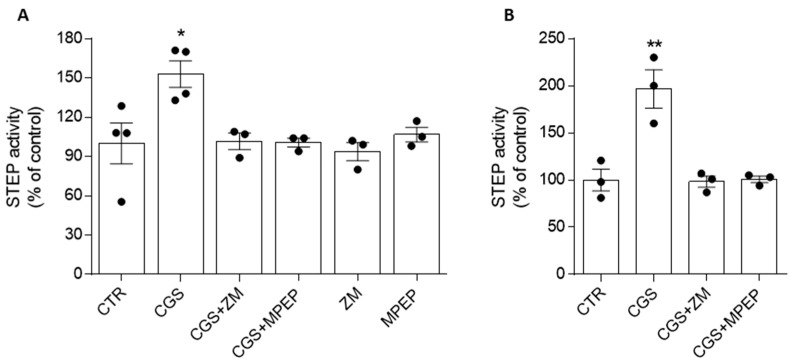
Modulation of A_2A_R affects STEP activity through the involvement of mGlu_5_R. (**A**) SH-SY5Y cells (n = 3/4 independent cell culture preparations) were treated for 5 min with CGS 21680 (CGS, 300 nM) alone, or with the A_2A_R antagonist ZM 241385 (ZM, 500 nM) or the mGlu_5_R antagonist MPEP (10 µM), which were added 20 min before and along with CGS. (**B**) Hippocampal slices (n = 3, each sample was obtained by pulling 8 slices) were treated for 20 min with CGS (300 nM) alone and pretreated with the A_2A_R antagonist ZM (500 nM) and the mGlu_5_R antagonist MPEP (10 µM), which were added 15 min before and along with CGS. The STEP activity was expressed as a percentage of the control (100%). The bar graphs represent the means + SEMs. * *p* < 0.05, ** *p* < 0.001 significantly different from control (one-way ANOVA followed by Tukey’s multiple comparison test).

We treated the SH-SY5Y cells with the selective A_2A_R agonist CGS 21680, and we evaluated the enzymatic activity of STEP in the immunocomplex obtained from the cell lysates using an anti-STEP antibody. In a previous paper, we demonstrated that the activity of STEP in the immunoprecipitate was almost completely abolished by treating the immunopellet with the STEP inhibitor TC-2153 1 µM, demonstrating that no other phosphatases co-precipitate with STEP [16]. As shown in Figure 1A, the application of 300 nM CGS 21680 for 5 min significantly increased STEP activity (153% ± 10% of the control, considered 100%, * *p* ≤ 0.05), an effect prevented not only by the A_2A_R antagonist ZM 241385 (500 nM) but also by the mGlu_5_R antagonist MPEP (10 µM) (Figure 1A). Notably, the treatments with ZM 241385 and MPEP alone did not change the STEP activity (Figure 1A).

We then confirmed the above results in mouse hippocampal slices: the treatment for 20 min with CGS 21680 (300 nM) increased the enzymatic activity of STEP (197% ± 20% of control, considered 100%, ** *p* ≤ 0.01), and this effect was hampered by a 15 min pretreatment with 500 nM of ZM 241385 or with 10 µM of MPEP (Figure 1B).

### 3.3. mGlu_5_R-Induced Increase in STEP Activity Is Prevented by the A_2A_R Antagonist

**Figure 2:**
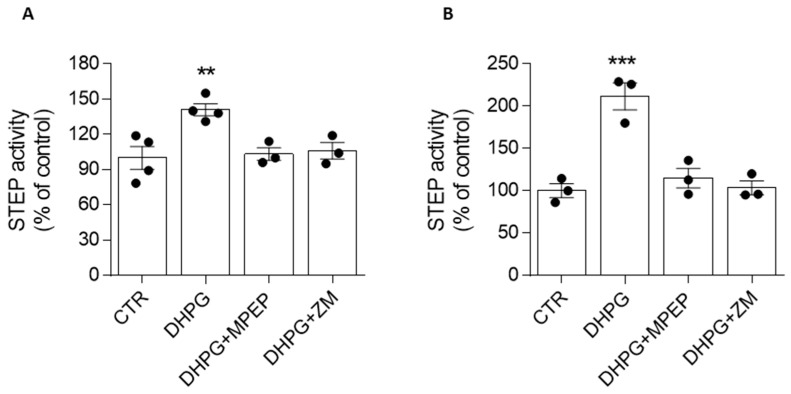
Modulation of mGlu_5_R affects STEP activity through the involvement of A_2A_R. (**A**) SH-SY5Y cells (n = 3/4 independent cell culture preparations) were treated for 5 min with DHPG (50 µM) alone and with the mGlu_5_R antagonist MPEP (10 µM) or the A_2A_R antagonist ZM 241385 (ZM, 500 nM), added 20 min before and along with DHPG. (**B**) Hippocampal slices (n = 3, each sample was obtained by pulling 8 slices) were treated for 10 min with DHPG (100 µM) alone, and with mGlu_5_R antagonist MPEP (10 µM) or A_2A_R antagonist ZM 241385 (500 nM), which were added 15 min before and then kept throughout the application of the DHPG. The bar graphs represent the means + SEMs. ** *p* < 0.01, *** *p* < 0.001 significantly different from the control (one-way ANOVA followed by Tukey’s multiple comparison test).

Five minutes of treating the SH-SY5Y cells with the mGlu_5_R agonist DHPG (50 µM) increased the enzymatic activity of STEP (141 ± 5% of control, considered 100%, ** *p* ≤ 0.01), and this effect was prevented by a 20 min pretreatment with the mGlu_5_R antagonist MPEP (10 µM) (Figure 2A). Interestingly, the DHPG-induced increase in STEP activity was also blocked by cell pretreatment with 500 nM of ZM 241385 (Figure 2A). The same results were obtained in the hippocampal slices, where we found that 100 µM of DHPG induced STEP activation (212 ± 16% of control, considered 100%, *** *p* ≤ 0.001), which was prevented not only by MPEP (10 µM) but also by ZM 241385 (500 nM) (Figure 2B).

### 3.4. Stimulation of A_2A_R and mGlu_5_R Induces STEP and mGlu_5_R to Physically Interact

**Figure 3:**
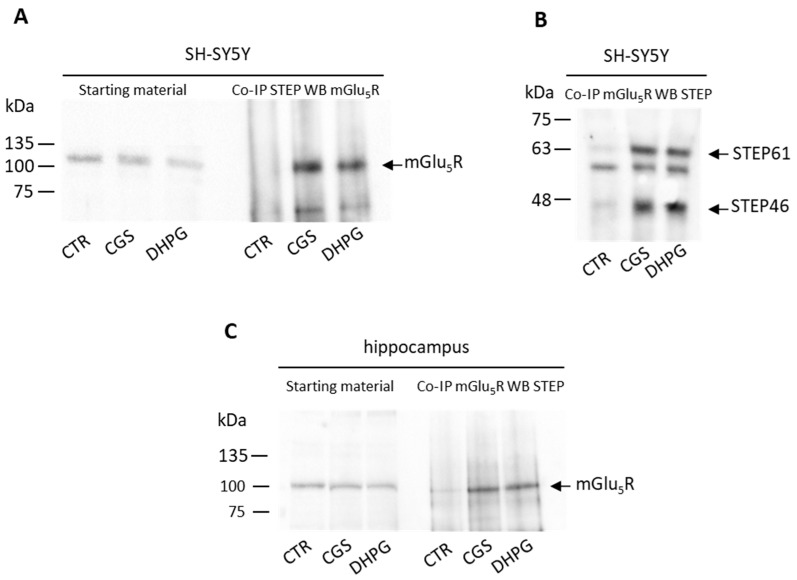
The stimulation of mGlu_5_ or A_2A_ receptors promotes the binding between STEP and mGlu_5_R. SH-SY5Y cells (**A**,**B**) and mouse hippocampal slices (**C**) were treated with the A_2A_R agonist CGS 21680 (CGS, 300 nM, 10 min) or with the mGlu_5_R agonist DHPG (50 µM, 10 min), and the Co-IP experiments were carried out using an anti-STEP monoclonal antibody (**A**,**C**) or an anti-mGlu_5_R polyclonal antibody (**B**). The presence of mGlu_5_R (**A**,**C**) or STEP (**B**) in the immunocomplexes was revealed via the relative polyclonal/monoclonal antibody. The immunoreactive bands were detected via chemiluminescence coupled to peroxidase activity (ECL). The molecular mass markers in kDa are indicated on the left. The starting materials in A and C (left panels) demonstrated that equal amounts of proteins were used. The blots displayed are representative of similar blots carried out in different preparations (n = 3). Original images can be found in Figure S3.

Since Won et al. [19] identified mGlu_5_R as a potential interactor with STEP, we wanted to verify whether STEP and mGlu_5_Rs could physically interact. To this aim, we performed a Co-IP assay in SH-SY5Y cells and mouse hippocampal slices in the control condition and after treatments with CGS 21680 or with DHPG (Figure 3). We immunoprecipitated STEP using the monoclonal antibody under experimental conditions that allow a protein–protein interaction to be maintained, and the presence of mGlu_5_R in the immunocomplex was revealed via a Western blot analysis, using an anti-mGlu_5_R polyclonal antibody. As shown in Figure 3A (right panel), in the SH-SY5Y cells, STEP and mGlu_5_R do not co-precipitate under control conditions, though they do after the cells are treated with CGS 21680 (300 nM) or DHPG (50 µM). The binding between STEP and mGlu_5_R also became evident if, conversely, we immunoprecipitated mGlu_5_R from SH-SY5Y using the polyclonal antibody, and the presence of the associated STEP was revealed via a Western blot analysis using the anti-STEP monoclonal antibody (Figure 3B). Interestingly, also in this case, the binding between STEP and mGlu_5_R was promoted via cell stimulation with the A_2A_ or mGlu_5_ receptor agonists (Figure 3B). The band below STEP61 can be explained as an aspecific signal of the monoclonal antibody since it does not change among the different conditions.

To further validate these results, we carried out the Co-IP assay in mouse hippocampal slices treated with 300 nM CGS 21680 or with 100 µM DHPG. The interaction between STEP and mGlu_5_R was evident when A_2A_R or mGlu_5_R were stimulated with the respective agonists (Figure 3C).

**Figure 4:**
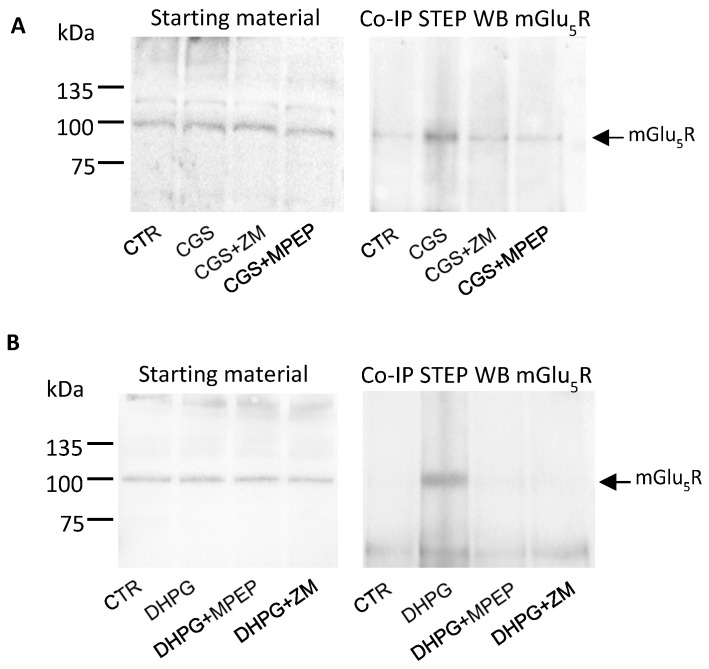
STEP/mGlu_5_R binding is prevented by A_2A_ and mGlu_5_ receptor antagonists. Hippocampal slices (each sample was obtained by pulling eight slices) were treated with CGS 21680 (CGS, 300 nM) (**A**) or with DHPG 10 µM (**B**) in the presence of the A_2A_R antagonist ZM 241385 (ZM, 500 nM) or mGlu5R antagonist (MPEP, 10 µM) applied 15 min before and along the application of CGS or DHPG. The interaction between STEP and mGlu5R was verified in the Co-IP experiments using a monoclonal anti-STEP antibody and the presence of mGlu5R in the immunocomplex was revealed by WB with a polyclonal anti-mGlu_5_R antibody. The immunoreactive bands were detected by chemiluminescence coupled to peroxidase activity (ECL). The starting materials in (**A**,**B**) (left panels) demonstrate that equal amounts of proteins were used. The molecular mass markers in kDa are indicated on the left. The blots displayed are representative of similar blots carried out in different preparations (n = 3). Original images can be found in Figure S4.

In hippocampal slices, the effect of CGS 21680 on promoting STEP/mGlu_5_R binding was blocked by pretreatment with the A_2A_R antagonist ZM 241385 (500 nM) or with the mGlu_5_R antagonist MPEP (10 µM) (Figure 4A), demonstrating that the effect of CGS 21680 was indeed A_2A_R-mediated, and that mGlu_5_R must be activated in order to interact with STEP. Notably, the effect of DHPG on mGlu_5_R/STEP Co-IP was prevented not only by MPEP but also by the A_2A_R antagonist ZM 241385 (Figure 4B), demonstrating that A_2A_R exerts a permissive role on mGlu_5_R/STEP interaction. The efficiency of STEP immunoprecipitation was evaluated via WB experiments, and we found that the STEP protein levels in the immunopellet did not change under the different conditions (Figure S5).

## 4. Discussion

This study demonstrates a close functional interaction between mGlu_5_ and A_2A_ receptors in the modulation of STEP activity. Indeed, our results show that: (i) A_2A_R-induced STEP activation requires the involvement of mGlu_5_R; (ii) A_2A_Rs must be activated in order to allow DHPG-induced STEP activation; (iii) the stimulation of mGlu_5_ and A_2A_ receptors drives mGlu_5_R to bind STEP and, possibly, to activate it.

Previously, we demonstrated that in the rat striatum and hippocampus, the stimulation of A_2A_R increases STEP phosphatase activity, and transgenic rats overexpressing the human A_2A_R showed increased basal STEP activity [16]. Using the neuroblastoma cell line SH-SY5Y, we could then demonstrate that the A_2A_R-induced activation of STEP was calcium-dependent and involved calcineurin activation [16]. The importance of raising intracellular calcium in A_2A_R-mediated effects was also highlighted by Gomez-Castro et al., who demonstrated the impact of Ca^2+^–calmodulin-activated adenylyl cyclases for the generation of cAMP [30]. The finding that A_2A_R stimulation increases STEP activity was somewhat unexpected since the stimulation of A_2A_R induced the activation of the cAMP/PKA pathway, which resulted in the direct inhibition of STEP activity through the phosphorylation of STEP at the serine residue and, indirectly, through the phosphorylation of DARPP-32 and the inhibition of PP1 [17,31,32]. Thus, the mechanism through which A_2A_R increased STEP activity must be independent from the cAMP/PKA pathway. The well-known interaction between the A_2A_ and mGlu_5_ receptors [7,11,20], together with the evidence that A_2A_R-mediated STEP activation is calcium-dependent, suggests mGlu_5_R as a possible mediator of A_2A_R’s effects on STEP activity. The results of the present study not only confirmed but also extended the hypothesis since it highlighted a close interdependence between the A_2A_ and mGlu_5_ receptors in the modulation of STEP activity. In fact, A_2A_Rs play a permissive role in DHPG-induced STEP activation since the blockade of A_2A_R does not allow DHPG to increase STEP activity. Moreover, we show that mGlu_5_R and STEP physically interact, as demonstrated via Co-IP experiments, and that their binding is promoted via A_2A_ and mGlu_5_ receptor stimulation. The first evidence that mGlu_5_R could be a STEP interactor came from the study of Won and collaborators, who used mass spectrometry to identify STEP61 binding proteins [19]. The study identified 315 candidate proteins, which included cytoskeletal-associated proteins, kinases and phosphatases, synaptic protein, and neurotransmitter receptors, including mGlu_5_R. To directly evaluate the binding of STEP to mGlu_5_R, we performed Co-IP assays using a STEP antibody incubated with lysates prepared from a mouse hippocampus or from neuroblastoma cells. Interestingly, while mGlu_5_R is poorly evident in STEP immunoprecipitates under control conditions, its presence is strongly increased after stimulation with A_2A_R and mGlu_5_R agonists. It is, thus, conceivable that a direct binding between STEP and mGlu_5_R is required in order to observe the activation of STEP induced by CGS 21680 or DHPG. The binding between STEP and mGlu_5_R is specifically mediated by A_2A_ and mGlu_5_ receptors since their selective antagonists strongly reduced the presence of mGlu_5_R in the STEP immunoprecipitates.

## 5. Conclusions

Given that the regulation of STEP has been implicated in the pathophysiology of a number of neurological and neuropsychiatric disorders [12], the interaction between A_2A_R and mGlu_5_R in the modulation of STEP activity appears to be particularly interesting since it could have a role in brain diseases [33] and could be involved in the therapeutic effects of A_2A_R and mGlu_5_R antagonists. Indeed, both A_2A_R and mGlu_5_R antagonists have been demonstrated to be effective in animal models of Alzheimer’s disease [11,34,35,36] and of fragile X syndrome [37,38,39,40], in which increases in the expression and activity of STEP have been demonstrated [12,37,41,42].

Our study thus further demonstrates the occurrence of cross-talk between A_2A_ and mGlu_5_ receptors, which must be taken into account for designing selective and efficacious therapeutics for the treatment of CNS diseases.

## Data Availability

The data that support the findings of this study are available from the corresponding author upon reasonable request.

## Supplementary Materials

The following supporting information can be downloaded at: https://www.mdpi.com/article/10.3390/biom13091350/s1, Figure S1: mGlu_5_R protein expression in SH-SY5Y neuroblastoma cells; Figure S2: CGS 21680 concentration response curves for cAMP accumulation recorded in SH-SY5Y cells stably expressing the cAMP 22 F sensor; Figure S3: Original images of Figure 3; Figure S4: Original images of Figure 4; Figure S5: STEP protein level in the immunocomplex after Co-IP with anti-STEP antibody.
