# Supplementary material for: Functional Interaction between Adenosine A2A and mGlu5 Receptors Mediates STEP Phosphatase Activation and Promotes STEP/mGlu5R Binding in Mouse Hippocampus and Neuroblastoma Cell Line
Source: Biomolecules. 2023 Sep 5;13(9):1350. doi: 10.3390/biom13091350 (PMC10527457; doi:10.3390/biom13091350)
Supplement: Supplementary file 1 [file biomolecules-13-01350-s001.zip › biomolecules-2565875-Supplementari Figure S1.pdf]

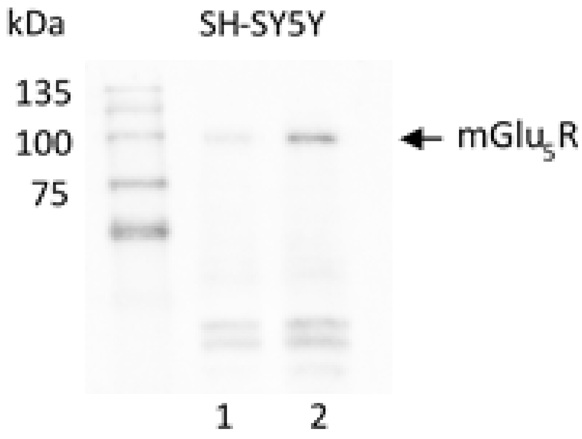

Figure S1.
mGlu5R protein expression in SH-SY5Y neuroblastoma cells. SH-SY5Y cells were lysed in RIPA buffer and proteins were transferred to nitrocellulose for western blot analysis. Lane 1 and lane 2 were loaded with 20 and 40 µg of total proteins, respectively. The blot was probed with monoclonal anti-mGlu5R antibody. The immunoreactive bands were detected by chemiluminescence coupled to peroxidase activity (ECL). The molecular mass markers in kDa are indicated on the left.
