# Supplementary material for: Functional Interaction between Adenosine A2A and mGlu5 Receptors Mediates STEP Phosphatase Activation and Promotes STEP/mGlu5R Binding in Mouse Hippocampus and Neuroblastoma Cell Line
Source: Biomolecules. 2023 Sep 5;13(9):1350. doi: 10.3390/biom13091350 (PMC10527457; doi:10.3390/biom13091350)
Supplement: Supplementary file 1 [file biomolecules-13-01350-s001.zip › biomolecules-2565875-Supplementary Figure S2.pdf]

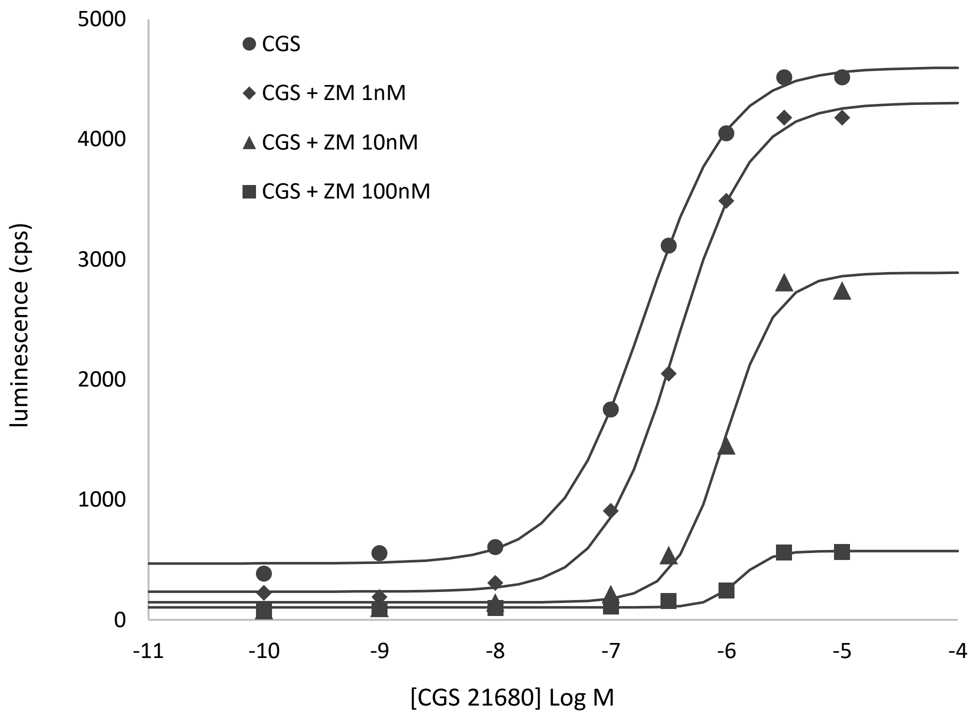

Figure S2

CGS 21680 concentration-response curves for cAMP accumulation recorded in SH-SY5Y cells stably expressing the cAMP 22F sensor

Each curve represents the increase of cAMP level as a function of CGS 21680 concentration alone or in combination with ZM 241385 at 1 nM, 10 nM or 100 nM, as indicated. Data points are means of triplicates obtained in one representative over three independent experiments. The solid lines are predicted from fits with a 4-parameter logistic model.

Fig S2

CGS concentration-response curves for cAMP accumulation recorded in SH-SY5Y cells stably expressing the cAMP 22F sensor

Each curve represents the increase of cAMP level as a function of CGS 21680 concentration alone or in combination with ZM 241385 at 1 nM, 10 nM or 100 nM, as indicated. Data points are means of triplicate obtained in one representative over three independent experiments. The solid lines are predicted from fits with a 4-parameter logistic model as described in vezzi et al (ref)

Materiali

cAMP measurements

cAMP assay was performed as described in (Vezzi V. et al., Scientific Reports 2020, 10:9111). Briefly, SH-SY5Y cells previously engineered for the stable expression of the cAMP GloSensor-22F probe (purchased from Promega), seeded a day before into 96-well white plates (Packard) were washed once with PBS and incubated for 60 min in 50 µl PBS containing 25 mM glucose and 2 mM luciferin (Oz Bioscience). Next, 50 µl PBS containing 100µM Rolipram with or without increasing concentrations of CGS 21680 alone, or in combination with ZM 241385 antagonist at either 1nM or 10nM or 100nM were added to the wells. Plates were immediately transferred into a luminometer (Victor Light, PerkinElmer). The total luminescence in each well (counts per second) was recorded at 30s intervals for 90 minutes with 0.5 s integration time.
