# Supplementary material for: Functional Interaction between Adenosine A2A and mGlu5 Receptors Mediates STEP Phosphatase Activation and Promotes STEP/mGlu5R Binding in Mouse Hippocampus and Neuroblastoma Cell Line
Source: Biomolecules. 2023 Sep 5;13(9):1350. doi: 10.3390/biom13091350 (PMC10527457; doi:10.3390/biom13091350)
Supplement: Supplementary file 1 [file biomolecules-13-01350-s001.zip › biomolecules-2565875-supplementary Figure S3-S5.pdf]

Figure S3

A

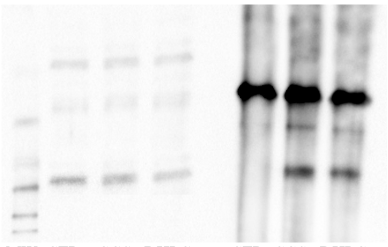

MW CTR CGS DHPG CTR CGS DHPG

B

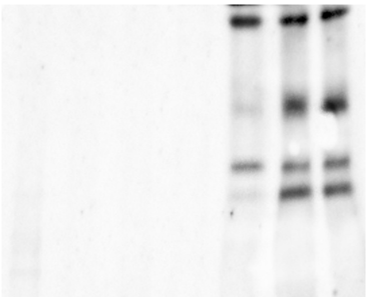

CTR CGS DHPG

C

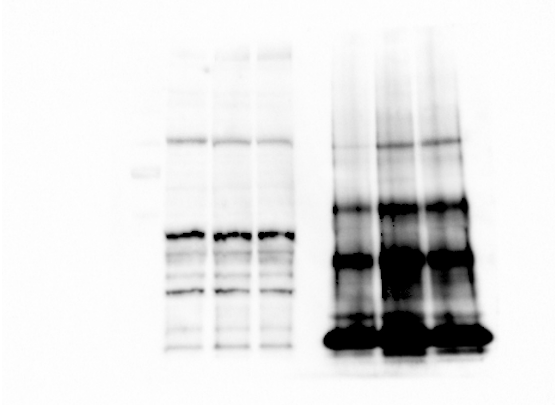

CTR CGS DHPG CTR CGS DHPG

Figure S4

A

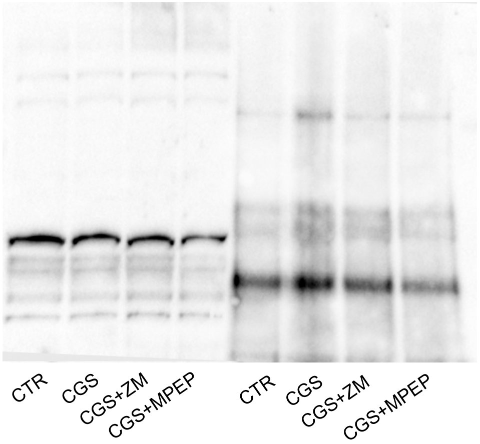
CTR
CGS
CGS+ZM
CGS+MPEP
CTR
CGS
CGS+ZM
CGS+MPEP

B

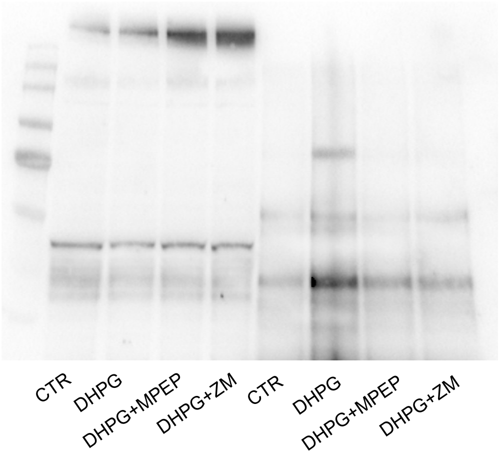
CTR
DHPG
DHPG+MPEP
DHPG+ZM
CTR
DHPG
DHPG+MPEP
DHPG+ZM

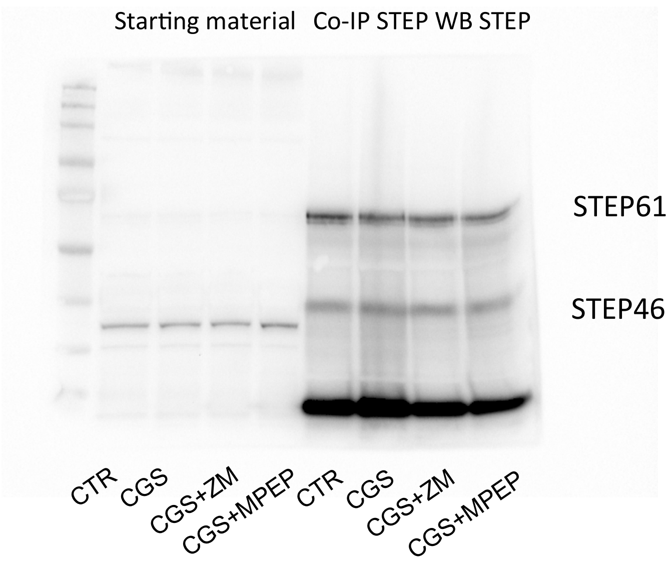

Figure S5
STEP protein level in the immunocomplex after Co-IP with anti-STEP antibody.
Hippocampal slices (each sample was obtained by pulling eight slices) were treated with CGS 21680 (CGS, 300 nM) in the presence of the A_2AR antagonist ZM 241385 (ZM, 500 nM) or $mGlu_5R$ antagonist (MPEP, 10 µM) applied 15 min before and along the application of CGS. The slices were lysed in RIPA buffer and the lysates were used for the Co-IP experiments using a monoclonal anti-STEP antibody. The level of STEP61 and STEP46 in the immunocomplex was verified by WB with a polyclonal anti-STEP antibody. The immunoreactive bands were detected by chemiluminescence coupled to peroxidase activity (ECL). The starting materials demonstrate that equal amounts of proteins were used. The blots displayed are representative of similar blots carried out in different preparations (n = 3).
